# Contribution of N^ε^-lysine Acetylation towards Regulation of Bacterial Pathogenesis

**DOI:** 10.1128/mSystems.00422-21

**Published:** 2021-08-24

**Authors:** Jackson Luu, Valerie J. Carabetta

**Affiliations:** a Department of Biomedical Sciences, Cooper Medical School of Rowan University, Camden, New Jersey, USA; Weill Cornell Medicine-Qatar

**Keywords:** posttranslational modification, acetylation, acetylome, virulence, pathogens, biofilm, antibiotic resistance, bacteria

## Abstract

N^ε^-lysine acetylation is an important, dynamic regulatory posttranslational modification (PTM) that is common in bacteria. Protein acetylomes have been characterized for more than 30 different species, and it is known that acetylation plays important regulatory roles in many essential biological processes. The levels of acetylation are enzymatically controlled by the opposing actions of lysine acetyltransferases and deacetylases. In bacteria, a second mechanism of acetylation exists and occurs via an enzyme-independent manner using the secondary metabolite acetyl-phosphate. Nonenzymatic acetylation accounts for global low levels of acetylation. Recently, studies concerning the role of protein acetylation in bacterial virulence have begun. Acetylated virulence factors have been identified and further characterized. The roles of the enzymes that acetylate and deacetylate proteins in the establishment of infection and biofilm formation have also been investigated. In this review, we discuss the acetylomes of human bacterial pathogens. We highlight examples of known acetylated virulence proteins and examine how they affect survival in the host. Finally, we discuss how acetylation might influence host-pathogen interactions and look at the contribution of acetylation to antimicrobial resistance.

## INTRODUCTION

**FIG 1:**
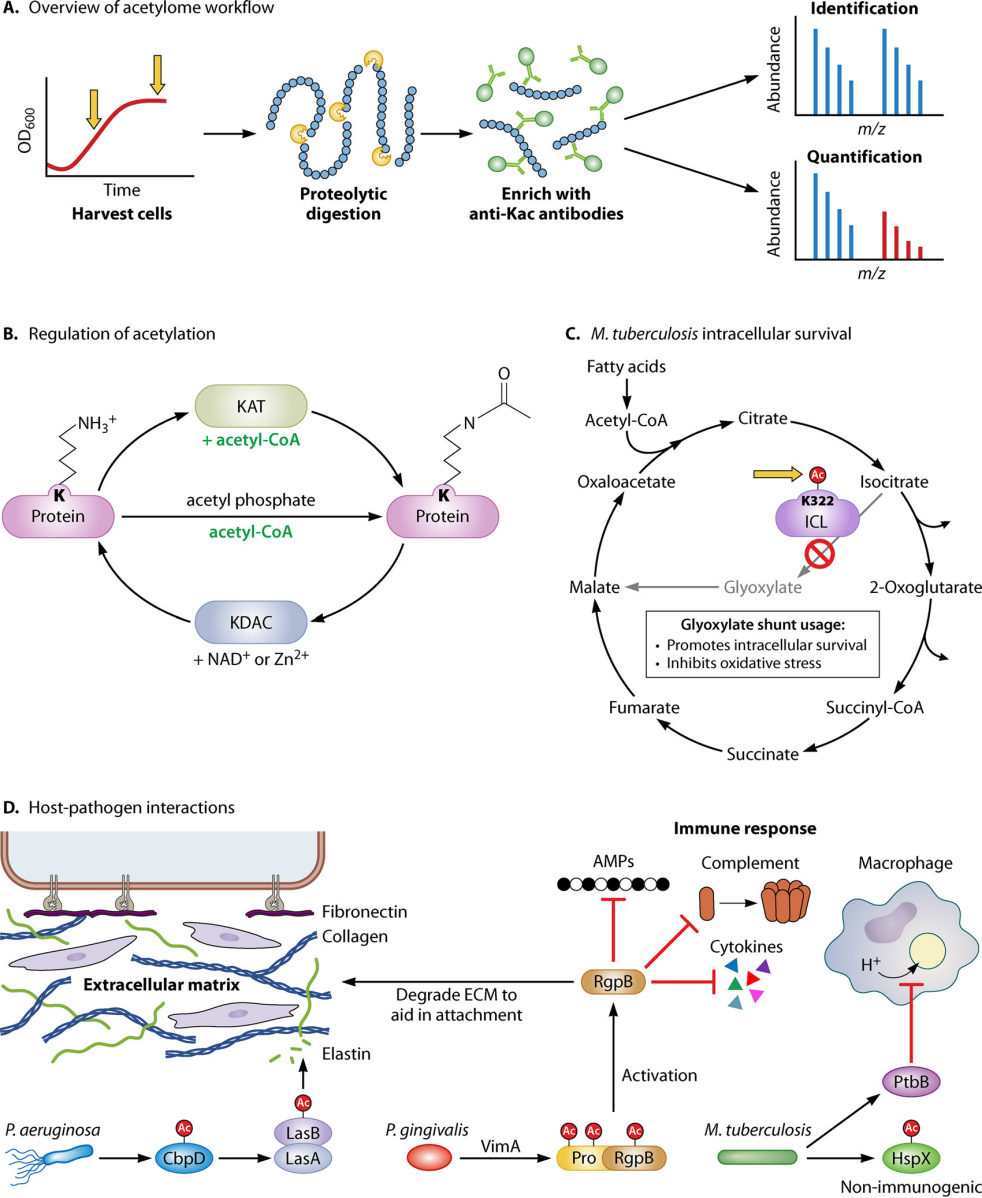
(A) Typical acetylome workflow. Cells are harvested at different growth phases or under different conditions, and proteins are digested, typically with trypsin. Often, acetylated peptides are enriched using anti-acetyllysine antibodies conjugated to agarose beads. The acetylated peptides are identified and quantified by MS. OD_600_, optical density at 600 nm. (B) Summary of the regulation of acetylation in bacteria. Proteins are acetylated either enzymatically by lysine acetyltransferases (KATs) or nonenzymatically via the high-energy intermediate acetyl-CoA or acetyl-phosphate. Deacetylation occurs by the action of NAD^+^-dependent sirtuins or Zn^2+^-dependent lysine deacetylases (KDACs). (C) For intracellular pathogens such as M. tuberculosis, intracellular metabolism is altered by acetylating key enzymes to control usage of the glyoxylate shunt. The glyoxylate shunt avoids the CO_2_-producing steps of the TCA cycle and replenishes intermediates. Acetylation of isocitrate lyase (ICL) at K322 inhibits its enzymatic activity and likely blocks usage of the shunt. Thus, deacetylation of ICL may be a critical step for intracellular survival. (D) Many secreted virulence factors are acetylated, which suggests that acetylation mediates pathogen-host interactions. P. aeruginosa secretes the acetylated proteins CbpD and LasB, which lead to the degradation of the human extracellular matrix (ECM) component elastin and aid in tissue invasion. In P. gingivalis, acetylation of the inactive pro-RgpB is required for enzyme activation as a protease. The acetylated pro-RgpB is secreted, where it is activated and degrades ECM components and immune system components, including cytokines, antimicrobial peptides (AMPs), and complement proteins. In M. tuberculosis, heat shock protein X is secreted and, when acetylated, is nonimmunogenic. The activity of the secreted protein tyrosine phosphatase PtpB is controlled by acetylation. PtpB promotes intracellular survival by inhibiting acidification inside the phagolysosome.

N^ε^-lysine acetylation is widely accepted as an important regulatory posttranslational modification (PTM) in bacteria. In the past decade, there has been an explosion of interest centered on protein acetylation in bacteria. Following the initial characterization of the Escherichia coli acetylome (1, 2), the acetylomes of more than 30 different species have been analyzed (reviewed in references 3–5). The typical workflow for acetylome analysis occurs in three main steps (Fig. 1A): (i) proteolytic digestion of proteins that were harvested following growth in a specific defined medium, (ii) enrichment of acetylated peptides through use of anti-acetyllysine antibodies, and (iii) identification or quantification of acetylation sites using mass spectrometry (MS)-based proteomics. The success of this workflow is largely due to the continuous improvements of MS-based technologies and the quality of anti-acetyllysine antibodies, enabling the identification of low-abundance acetylation sites and a growing number of identified acetylated proteins (3, 4).

In bacteria, lysines are acetylated by two different mechanisms, enzymatic and nonenzymatic (Fig. 1B). Bacterial lysine acetyltransferases (KATs) are members of the Gcn5 N-acetyltransferase (GNAT) family that catalyze the addition of an acetyl group from acetyl-CoA (Ac-CoA) to a target lysine residue. This action is opposed by lysine deacetylases (KDACs), mostly members of the NAD^+^-dependent sirtuin family (5). Nonenzymatic acetylation by the high-energy intermediate acetyl-phosphate (AcP) and, possibly, Ac-CoA occurs at a low level when the local environment surrounding target lysine residues is favorable. Nonenzymatic acetylation is dependent upon intracellular AcP levels, which are controlled by the coordinated action of the enzymes phosphotransacetylase (Pta) and acetate kinase (AckA). Pta catalyzes the reversible reaction to convert Ac-CoA to AcP, and AckA catalyzes the reversible reaction to convert AcP to acetate (reviewed in reference 6). In Escherichia coli, it was demonstrated that global acetylation occurs via this nonenzymatic mechanism (7, 8). This finding is also true in Bacillus subtilis, Neisseria gonorrhoeae, and possibly all bacteria (9, 10). Nonenzymatic acetylation can be reversible, as it was shown in E. coli that the sirtuin CobB can deacetylate lysine residues irrespective of the mechanism of acetylation. However, while CobB is the major deacetylase in E. coli, only a small fraction of sites are subjected to this regulation and are reversible (11). Because two recent reviews have been published (5, 12), the mechanisms of acetylation will not be covered further here.

The next step for the bacterial acetylation field is to focus on understanding the physiological relevance of the thousands of identified acetylation sites to determine which of them represent important regulatory events. To study the effects of acetylation *in vivo*, lysine is commonly mutated to glutamine to mimic the acetylated state and to arginine to mimic the unacetylated state, which maintains the positive charge but cannot be acetylated. In addition, MS techniques are being developed to investigate the proportion of acetylation occupancy, or stoichiometry (4, 13–18), which is crucial to help prioritize further investigations. Currently, essential processes have been the subject of such investigations. For example, extensive work has been done assessing the role of acetylation in controlling cellular metabolism in response to environmental cues (reviewed in references 5 and 12). In addition, the functional relevance of lysine acetylation of selected individual proteins is being explored. The chemotaxis regulatory protein CheY, the regulator of capsule synthesis B (RcsB), and acetyl-CoA synthetase (Acs; reviewed in references 5 and 12) were some of the first proteins with identified functional roles for lysine acetylation (19–28). One emerging focus has been on investigating acetylated proteins involved in bacterial virulence, a topic that was recently reviewed in 2017 (29). The contribution of CheY and RcsB acetylation to bacterial virulence has been previously reviewed (12, 29) and will not be discussed here. In this review, we will first highlight new examples of how acetylated proteins contribute to bacterial survival in the host, including in the presence of antibiotics. We will then discuss how acetylation of bacterial proteins may influence interactions with host proteins. Finally, we will end with a discussion on how we can use this information to design novel treatment strategies to deal with troublesome infections.

## ACETYLOME ANALYSIS IN PATHOGENIC BACTERIA

The characterization of the acetylomes of clinical pathogens has increased significantly in recent years, including in Staphylococcus aureus (30), Borrelia burgdorferi (31), Leptospira interrogans (32), and Acinetobacter baumannii (33). Some acetylome analyses revealed important acetylated virulence factors. The acetylated proteins involved in the virulence of A. baumannii were discussed in a prior review (29). Recently, it was found that the virulence of Streptococcus pneumoniae also might be regulated by acetylation (34). Seventeen proteins that are known virulence factors were acetylated, including enzymes involved in capsular polysaccharide biosynthesis. Production of capsule is a mechanism for immune evasion, and acetylation may be important to modulate this process (35). Vibrio cholerae contains 68 acetylated proteins that are known virulence factors (36), including important transcriptional regulators, such as AphB and LuxU. Structural examination of AphB predicted that acetylation site K103 lies in the dimerization interface and may influence protein-protein interactions (36). For the phosphorelay protein LuxU, acetylation occurred on K53, which is physically close to the phosphoacceptor site (H57), and acetylation was proposed to impact the phosphorylation state. However, none of these predictions have been experimentally confirmed; therefore, the physiologic significance of these observations remains unclear. The acetylomes of other pathogenic *Vibrio* species have also been characterized, including V. vulnificus (37), V. parahaemolyticus (38), and V. alginolyticus (39), but investigation of potential virulence factors was not extensively explored.

For the majority of these species, the acetylome characterizations were performed in defined chemical media under laboratory conditions. To fully understand how acetylation influences bacterial virulence, these studies on specific virulence factors must be performed in tissue culture or animal infection models. The challenge will be obtaining enough material to accurately quantify the acetylome, but the currently available mass spectrometers with the latest technological improvements should make this possible.

## THE ROLE OF ACETYLATION ON INTRACELLULAR SURVIVAL

Virulence factors are proteins or other substances that are required for establishment of infection, acquisition of nutrients in the host environment, and immune evasion. For the remainder of this review, we will discuss examples of acetylated virulence factors with experimental validation (Table 1). Various large-scale acetylome analyses have been performed in Mycobacterium tuberculosis, and numerous acetylated proteins have been linked to virulence (40–42). Xie et al. found 20 acetylated proteins that were involved in virulence (40). One of these proteins is the metabolic enzyme isocitrate lyase (ICL1). During latent infection, M. tuberculosis alters carbon metabolism and relies on fatty acids from the host as their predominant carbon source (43). Some fatty acids are oxidized to produce Ac-CoA, which then enters the tricarboxylic acid (TCA) cycle by using the glyoxylate shunt, where ICL1 is involved (Fig. 1C). The glyoxylate shunt is primarily utilized to assimilate carbon when the source is 2C or 3C in length, such as acetate, which bypasses the CO_2_ generating steps of the TCA cycle and replenishes intermediates for biosynthetic processes (44). Thus, *icl* mutants cannot grow on fatty acids and display attenuated virulence in mouse infection models (45, 46). *ICL1* is acetylated at K322, and mutation of this site to glutamine to mimic the acetylated state resulted in a decrease of enzymatic activity (40, 42). Therefore, deacetylation may be an important mechanism to regulate a shift toward usage of the glyxoylate shunt during host infection (Fig. 1C).

**TABLE 1:** Acetylated virulence factors^*a*^

Species	Acetylated protein	Functional acetylation site	Function	Consequence of acetylation	Reference(s)
*A. hydrophila*	LuxS	K165	Production of AI-2	Inhibits enzymatic activity	57
B. pertussis	BvgA	Multiple	Response regulator, virulence expression	Unknown	48
E. coli	CheY	92, 109	Chemotaxis and motility regulator	Alters interactions, promotes CW rotation	22, 25–27
	RcsB	154, 180	Regulates capsule synthesis and biofilm	Inhibits DNA binding	19 – 21
M. tuberculosis	ICL1	K322	Central metabolism, glyoxylate shunt	Inhibits enzymatic activity	40, 42
	PtpB	K224	Protein tyrosine phosphatase	Inhibits enzymatic activity	81
	DosR	K182	Response regulator induced by hypoxia	Inhibits DNA binding	54
	MtrA	K110	Repressor of cell division	Inhibits DNA binding	49, 50
	HspX	Multiple	Heat shock protein X, immunogenic	Decreases immunogenicity	41
P. gingivalis	RgpB	Multiple	Cysteine protease, required for host survival	Required for processing to mature, active enzyme	76
	RprY	Unknown	Response regulator, controls expression of the T9SS	Inhibits DNA binding and reduces phosphorylation	77
S. mutans	GtfB, GtfC, GtfD	Multiple	Glucan synthesis, biofilm formation	Decreases enzymatic activity	61
P. aeruginosa	LasB	Multiple	Elastase, degrades elastin	Unknown	64
	CpbD	Multiple	Chitin binding protein, staphylolytic activity	Unknown	64
F. novicida	ChiA and ChiB	Multiple	Chitinases	Inhibits activity, promotes biofilm	71
*S.* Typhimurium	PhoP	201	Response regulator, responds to low Mg^2+^ or acidic pH	Inhibits DNA binding	91
	HilD	297	Transcription factor, regulates SPI-1	Increases stability, reduces DNA binding	92
	AcrB	K1037	Multidrug efflux pump	Regulates activity of pump	82

a Abbreviations: AI-2, autoinducer-2; CW, clockwise; T9SS, type IX secretion system; SPI-1, Salmonella pathogenicity island 1.

## THE ROLE OF ACETYLATION ON TRANSCRIPTIONAL FACTORS THAT REGULATE VIRULENCE

One area that has been extensively studied is the role of acetylation in the modulation of transcription factor activity, typically by blocking DNA binding activity. Some examples that have been previously reviewed (13) are the acetylation of the transcription factors HilD and PhoP in Salmonella enterica serovar Typhimurium. In Bordetella pertussis, BvgA, a response regulator of the BvgAS two-component system (47), contains eight acetylation sites (48). It was determined that acetylation of BvgA does not alter protein levels or phosphorylation state; however, the effect on DNA binding was not assessed (48). Since at least two acetylation sites occur in the helix-turn-helix DNA binding domain, it is likely that acetylation inhibits the DNA binding activity of BvgA, similar to what was observed for other transcription factors. It would be interesting to generate glutamine substitutions to confirm this suggestion and determine the effect of such mutations in *in vivo* mouse models.

In M. tuberculosis H37Rv, MtrA of the MtrAB two-component system (49, 50) enhances its interaction with its cognate sensor kinase MtrB and reduces DNA binding activity. The phosphorylated form of MtrA is a repressor that sequesters *oriC* to block cell division and blocks expression of resuscitation promoting factor (Rpf), which hydrolyzes peptidoglycan during cell division and is required for reactivation (51–53). Therefore, active MtrA leads to latency. Acetylation of K111, which is located in the N-terminal receiver domain, was proposed to be a mechanism to turn off the MtrA repressor, possibly in response to nutrient availability, which would allow for resumption of bacterial growth and escape from latency. Interestingly, DosR, another response regulator involved in latency control, is regulated in the same fashion (54). Acetylation of DosR, which is induced under hypoxic conditions, is acetylated at K182, which inhibits DNA binding. DosR activates expression of 48 genes that are required for survival during latency (55). Because it has been demonstrated that the sirtuin Rv1151c deacetylates DosR and MtrA, drugs that specifically target Rv1151c may prevent the development of latent tuberculosis infections.

## THE ROLE OF ACETYLATION DURING BIOFILM FORMATION

The majority of bacterial clinical infections are biofilm based, owing to the fact that biofilms offer bacteria protection from host immune responses and antibiotics (56). One protein involved in biofilm formation that was identified as acetylated in Aeromonas hydrophila is the *S*-ribosylhomocysteine lyase LuxS, which is involved in production of the quorum-sensing molecule autoinducer-2 (57, 58). LuxS is acetylated at K165, and, interestingly, this site is also succinylated (57). Acetylation of K165 inhibits enzymatic activity, while succinylation has the opposite effect, and the sirtuin CobB removes both modifications. In addition, a *luxS* deletion mutant exhibits enhanced biofilm production and increased virulence in mouse models (59). Taken together, acetylation of LuxS decreases enzymatic activity, which in turn may enhance biofilm formation and, therefore, virulence. To test this prediction, the biofilm properties and effects on virulence in a mouse model of *luxSK165Q* and *cobB* deletion strains should be determined. Identification of the LuxS acetylation mechanism may represent a new drug target to limit biofilm-based infections.

Streptococcus mutans produces plaque biofilms that contain glucans, which allow for adherence to the tooth surface (60). The three major glucosyltransferase enzymes, GtfB, GtfC, and GtfD, synthesize glucans and have decreased acetylation levels during biofilm growth compared to free-living, planktonic bacteria. Moreover, the enzymatic activity of these enzymes increases during biofilm growth, suggesting that acetylation is a mechanism to turn off glucan biosynthesis (61) when bacteria are not present in biofilms.

Increasing global acetylation often leads to large alterations in biofilm properties. For example, in M. tuberculosis H37Ra, an attenuated strain, deletion of the known sirtuin (MRA_1161, an ortholog of Rv1151c) results in defective biofilm formation (41), possibly due, in part, to inhibition of fatty acid metabolic enzymes via acetylation of key lysine residues in their active sites (62). In Neisseria gonorrhoeae, an acetate kinase mutant (*ackA*) displayed marked defects in maintaining biofilm structure over time (10). AckA is an enzyme involved in the reversible conversion of AcP to acetate, and in its absence, AcP accumulates (6, 12). As AcP is the main acetyl donor for nonenzymatic acetylation (12), this suggests that key biofilm regulatory proteins are increasingly acetylated, which leads to the observed defects. Further analysis is required to determine which important biofilm regulatory proteins are acetylated in N. gonorrhoeae.

## THE ROLE OF ACETYLATION DURING INTERACTION WITH THE HOST

Proteins that are secreted often interact with the host to invade cells and destroy immune cells or other antimicrobial products (Fig. 1D). In Pseudomonas aeruginosa, several virulence factors were identified as acetylated or succinylated, including components of secretion systems and secreted factors (63). For example, two secreted virulence factors, CbpD and LasB, were found to be modified by multiple different PTMs in the extracellular environment (64). CbpD is a chitin binding protein with staphylolytic activity and is needed to process and activate LasA, which is involved in the degradation of the extracellular matrix protein elastin (65, 66). LasA enhances the elastase activity of LasB (67), which leads to tissue destruction in the host. Nine different lysine modifications, including acetylation, butyrylation, crotonylation, di- and tri-methylation, malonylation, methylation, propionylation, and succinylation, were identified in these proteins in the extracellular environment (64), but the effect of these PTMs on enzymatic activity or protein-protein interactions is not known. The mechanism of acylation is also unclear, as it may occur intracellularly or extracellularly by an acyltransferase or a nonenzymatic mechanism. This raises the interesting possibility that protein speciation, the presence of multiple PTMs on a single protein arising from one gene (68, 69), gives a broad functional diversity to a limited number of secreted proteins in the host environment, referred to as moonlighting (68, 70), information that may be essential for our understanding of bacterial virulence.

Francisella novicida contains 12 acetylated secreted proteins that are essential for proliferation and survival (71). Two chitinases, ChiA and ChiB, were among those secreted proteins. *In vitro* chemical acetylation with AcP resulted in a decrease in chitinase activity. These enzymes negatively regulate biofilm formation (72), so acetylation may inhibit chitinase activity to promote biofilm formation, aiding in host colonization. Acetylome profiling of Porphyromonas gingivalis revealed numerous interesting acetylated secreted proteins that play a role in bacterial virulence (73). A major group of virulence factors is the gingipains, which are secreted cysteine proteases that are required for invasion of tissues, inactivation of cytokines, and acquisition of essential nutrients (74). The gingipains consist of an arginine-specific protease (encoded by *rgpA* and *rgpB*) and a lysine-specific protease (75). The inactive, preprocessed proenzyme of RgpB (pro-RgpB) is acetylated by VimA and its paralog, PG1842. This acetylation is required for enzyme activation and, therefore, virulence (76). The transcription factor RprY, an orphan response regulator, was shown to be enzymatically regulated by the KAT Pat and sirtuin CobB, which inhibits its DNA binding activity and reduces phosphorylation (77). RprY regulates the expression of the type IX secretion system (T9SS), which exports many virulence factors. In support of this, RprY is required for virulence in a murine model (78). Thus, inhibition of RprY DNA-binding by acetylation is a mechanism to directly control secretion of virulence factors.

In M. tuberculosis, 45 secreted proteins were found to be acetylated, many of which stimulate host immune responses. Heat shock protein X (HspX) is secreted and has been shown to stimulate interferon gamma production in the host (79). Interestingly, blood samples from tuberculosis patients were collected and used to test the immunogenicity of acetylated and unacetylated fragments of HspX. The immune responses to the acetylated form of the protein were much weaker or absent (41). Therefore, acetylation of secreted proteins may be a way for bacteria to alter the host immune response and increase intracellular survival. Another secreted protein is the protein tyrosine phosphatase PtpB, which dephosphorylates various host proteins and is responsible for promoting intracellular survival by inhibiting acidification inside the phagolysosome (80). Acetylation of PtpB is controlled by the KAT (Pat) and deacetylase Rv1151c, and acetylation at site K224 decreases the rate of the phosphatase reaction (81). Again, Rv1151c may be an attractive drug target, as its inhibition would increase acetylation of PtpB and may have significant phenotypic consequences on downstream host targets, which may lead to less intracellular survival.

## THE ROLE OF ACETYLATION IN ANTIBIOTIC RESISTANCE

The role of lysine acetylation in antibiotic resistance has not been well studied, but hints of its importance are emerging. For example, in Salmonella Typhimurium, 15 resistance-related proteins were acetylated, including a multidrug efflux transporter (AcrB) and various outer membrane proteins (OMPs), which may decrease outer membrane permeability (82). Following the development of fluoroquinolone resistance, the acetylation abundance increased on some OMPs and decreased on AcrB, suggesting that acetylation regulates the activity of these proteins and contributes to resistance development. In M. smegmatis, the histone-like protein HupB is acetylated at multiple sites. Mutation of K86 to arginine (unmodified mimic) resulted in the specific loss of the small-colony variant, isoniazid-tolerant subpopulation (83). As discussed before, in M. tuberculosis the metabolic enzyme isocitrate lyase (ICL) is acetylated. When exposed to isoniazid, rifampin, and streptomycin, the ICL enzymes are activated (84). Indeed, *icl* mutants are 100- to 1,000-fold more susceptible to these drugs, and this can be rescued by growth in the presence of an antioxidant, such as thiourea. This suggests that the ICLs participate in the defense against antibiotic-induced oxidative stress; therefore, deacetylation of ICL may be critical for drug tolerance.

## CONCLUDING REMARKS

The physiological relevance of protein acetylation in bacteria is an important topic that is just beginning to be addressed, as there remain thousands of uncharacterized sites. So far, many important basic biological processes are regulated by acetylation, and the initial focus has largely been on transcription, translation, and metabolism (3–5, 12, 29). Recently, we have begun to investigate virulence, but there is still much to discover. Large-scale acetylome analysis can reveal hints of additional virulence proteins to investigate. For example, in E. coli the bifunctional acetaldehyde-CoA dehydrogenase and alcohol dehydrogenase AdhE was identified as acetylated at >10 sites in multiple studies (1, 7, 8, 13, 14, 17, 19, 85, 86). AdhE regulates host-cell binding and establishment of infection of enterohemorrhagic E. coli, and an *adhE* mutant displays attenuated virulence in rabbit models of infection (87). Therefore, AdhE acetylation may be an essential regulatory mechanism for survival in the host and is worthy of further investigation.

Our understanding of how acetylation regulates virulence factors could aid in the design of novel therapeutics. In theory, we could design drugs that specifically target the known enzymes of acetylation, either KATs, KDACs, or even acetate kinase, which could alter the levels of acetylation and limit virulence. For example, the M. tuberculosis sirtuin Rv1151c is a promising target. Inhibiting this enzyme may affect the establishment of M. tuberculosis latency by increasing the acetylation of MtrA and DosR (49, 54) while also decreasing the activity of the secreted phosphatase PtpB (81), mitigating intracellular survival. Recently, it was demonstrated that using a combination of the antibiotic fusidic acid followed by a KAT inhibitor (EIS 1a*) led to increased killing of M. smegmatis compared to antibiotic alone, a strategy that could be optimized for use with M. tuberculosis (88).

Interestingly, many secreted factors are modified by acetylation, but it is unclear if they are modified in the cytoplasm before secretion or in the extracellular environment. Some N_α_-acetyltransferases (NATs), which acetylate the amino group of the N terminus of proteins, are known to be secreted. For example, in M. tuberculosis, members of the ESAT6 family of NATs are critical virulence factors and are present in the extracellular environment (89, 90). Currently, there are no examples of extracellular bacterial KATs or KDACs. However, if it turns out that extracellular KATs and KDACs do exist, it is intriguing to think of the possibility of designing drugs that block interactions with these enzymes to possibly interfere with host-pathogen interactions. This could make an infection less severe or easier for the immune system to clear. Because emerging evidence indicates that acetylation is involved in the development of antibiotic resistance, inhibiting acetylation may become a weapon to control the emergence of drug-resistant bacteria. As we learn more about the contributions of acetylation toward bacterial pathogenesis, exciting new avenues for novel therapeutics will emerge.
